# S100A6 Promotes B Lymphocyte Penetration Through the Blood–Brain Barrier in Autoimmune Encephalitis

**DOI:** 10.3389/fgene.2019.01188

**Published:** 2019-11-22

**Authors:** Meng-Han Tsai, Chih-Hsiang Lin, Kuo-Wang Tsai, Ming-Hong Lin, Chen-Jui Ho, Yan-Ting Lu, Ken-Pen Weng, Yuyu Lin, Pei-Hsien Lin, Sung-Chou Li

**Affiliations:** ^1^Department of Neurology, Kaohsiung Chang Gung Memorial Hospital and Chang Gung University College of Medicine, Kaohsiung, Taiwan; ^2^Department of Medical Education and Research, Kaohsiung Veterans General Hospital, Kaohsiung, Taiwan; ^3^Department of Microbiology and Immunology, School of Medicine, College of Medicine, Kaohsiung Medical University, Kaohsiung, Taiwan; ^4^Research Center for Environmental Medicine, Kaohsiung Medical University, Kaohsiung, Taiwan; ^5^Department of Pediatrics, Kaohsiung Veterans General Hospital, Kaohsiung, Taiwan; ^6^Department of Medicine, National Yang-Ming University, Taipei, Taiwan; ^7^Department of Nursing, Shu-Zen Junior College of Medicine and Management, Kaohsiung, Taiwan; ^8^Genomics and Proteomics Core Laboratory, Kaohsiung Chang Gung Memorial Hospital and Chang Gung University College of Medicine, Kaohsiung, Taiwan

**Keywords:** autoimmune encephalitis, DNA methylation, S100A6, leukocyte transendothelial migration, B cell infiltration, blood–brain barrier

## Abstract

Autoimmune encephalitis (AE) is a severe neurological disease. The brain of the AE patient is attacked by a dysregulated immune system, which is caused by the excessive production of autoantibodies against neuronal receptors and synaptic proteins. AE is also characterized by the uncontrolled B lymphocyte infiltration through the blood–brain barrier (BBB) layer, and the investigation of the underlying mechanism involved in this infiltration may facilitate the discovery of novel therapies for AE. However, few AE-related studies have focused on this issue. In this study, we aimed to identify the factors involved in B lymphocyte infiltration in AE. For this purpose, we first enrolled four healthy control and five AE subjects, collecting their serum and/or total white blood cell samples. The white blood cell samples were further used for collecting RNA and DNA. Then, we simulated the *in vivo* B lymphocyte infiltration with an *in vitro* leukocyte transendothelial migration model. It turned out that AE serum treatment significantly and specifically promoted B cells to penetrate the BBB endothelial layer without affecting neutrophils. Next, through genome-wide DNA methylation assays on bisulfite-conversion DNA samples, we identified S100A6 and S100A11 as potential hypo-methylated disease genes in the AE samples. Further qPCR assays demonstrated their upregulation in AE samples, reflecting the negative correlations between gene expression and DNA methylation. Finally, recombinant S100A6 protein treatment significantly increased B lymphocyte infiltration through the BBB endothelial layer, which partially recapitulated the effect of AE serum. In summary, by using an *in vitro* leukocyte transendothelial migration model, we confirmed that S100A6 promoted B lymphocyte to penetrate the BBB endothelial layer, which is similar to the *in vivo* clinical manifestations of AE. Therefore, further studies on how the S100A6 protein facilitates B lymphocyte infiltration and on whether other factors in serum also contribute to this phenomenon are likely to improve our understanding of AE and hopefully to reveal novel therapeutic targets for this emerging treatable neurological disorder.

## Introduction

Autoimmune encephalitis (AE) is a severe neurological disease in which the brain becomes the target of dysregulated immune responses (Newman et al., 2016). In the past decade, AE cases have been increasingly recognized due to the discovery of neuron-specific autoantibodies in the blood and/or cerebrospinal fluid (CSF) (Dubey et al., 2016). The pathogenic role of antibodies has been demonstrated by the passive transfer of autoantibodies to animal models, which recapitulated the effects of autoantibodies and the clinical manifestations of AE (Planaguma et al., 2015; Wright et al., 2015). However, the mechanisms through which these autoantibodies are generated and enter the central nervous system (CNS) remain elusive. The interactions between genetic predisposition and environmental factors have long been proposed to play a role in AE and other autoimmune disorders. In addition, several HLA alleles have been reported to be associated with AE (Kim et al., 2017; van Sonderen et al., 2017; Mueller et al., 2018).

The clinical manifestations of AE are not specific and they include fever, behavior changes, memory loss, psychosis, seizures, and dyskinesias (Dubey et al., 2015; Graus et al., 2016). Therefore, delayed or inappropriate initial management is not uncommon (Armangue et al., 2014; Ellul and Solomon, 2018). The diagnosis of AE relies on clinical symptoms, autoimmune tests, and, sometimes, the responses to immunotherapy (Zuliani et al., 2012; Graus et al., 2016). Although consensus diagnostic criteria (Graus et al., 2016) have been proposed for AE, the similarities of clinical symptoms between AE and infectious encephalitis frequently make initial clinical differentiation a challenging task (Lin et al., 2019). The autoimmune tests were limited by their availability and the time required for the results. Furthermore, a negative result for currently known autoantigen examination does not exclude the diagnosis of AE (Graus et al., 2016). In addition, the responsiveness to immunotherapy was not specific because other disorders may also respond to immunotherapy, such as primary CNS lymphoma (Graus et al., 2016). Therefore, identifying novel biomarkers could be helpful for the diagnosis of AE and may facilitate the clinicians’ decision making to initiate the prompt immunotherapy.

DNA methylation is a common epigenetic mechanism that regulates the transcriptional activity of gene expression. It plays important roles in the pathogenesis mechanism of many autoimmune diseases (Wang and Lu, 2017) such as systemic lupus erythematosus, rheumatoid arthritis, and multiple sclerosis (Sun et al., 2016). The differential DNA methylation in these diseases regulated the disease-related genes, controlling disease outbreak. And, sometimes it is also involved in disease severity. AE is characterized by autoantibodies and B cell infiltration through the blood–brain barrier (BBB) layer (Gastaldi et al., 2016). Even with these previous AE-related studies, the pathogenesis mechanism of AE remains unclear. In other words, what elements drive B cell infiltration? In addition, why such B cell infiltration takes place only at the BBB endothelial layer? To answer our questions, we first conducted an *in vitro* leukocyte transendothelial migration (LTEM) assay to mimic AE symptoms that the serum from AE patients promoted B cell infiltration through BBB endothelial layer. Then, by analyzing genome-wide DNA methylation data, we identified S100A6 and S100A11 as the candidate disease genes of AE. Using the LTEM assay as an *in vitro* cell model, we further confirmed that S100A6 promoted B cells to penetrate BBB endothelial layer, causing the phenomenon similar to the *in vivo* observations in AE patients.

## Materials and Methods

### Subject Enrollment

We enrolled five AE patients and four healthy control (HC) subjects for this study in Kaohsiung Chang Gung Memorial Hospital. The AE patients were diagnosed according to the following criteria as suggested by previous experts’ opinions (Graus et al., 2016):

1. Sub-acute onset (rapid progression of fewer than 3 months) of working memory deficits (short-term memory loss), altered mental status, or psychiatric symptoms.
2. At least one of the following symptoms:
  - New focal CNS findings
  - Seizures not explained by a previously known seizure disorder
  - CSF pleocytosis (white blood cell count of more than five cells per mm^3^)
  - MRI features suggestive of encephalitis
3. Reasonable exclusion of alternative causes

In addition, the serum and/or CSF samples of all AE patients have been tested for common autoantibodies, including anti-nuclear antibodies, anti-dsDNA, anti-extra nuclear antigens, and anti-thyroid autoantibodies as well as a commercialized cell-based assay for antibodies against neuronal surface antigens (Autoimmune Encephalitis Mosaic 6 Assay, EUROIMMUN, Germany). Patients who were negative for neuronal surface antigen antibodies were diagnosed as AE due to unknown antigen. All patients with AE due to unknown antigen required responsiveness to immunotherapy to be included in this study.

### Sample Collection, Bisulfite Conversion, and Methylation Assay

The AE patients were subject to peripheral blood collection before immunotherapy, followed by serum and blood cell enrichment. The same collection procedures were also applied on HC subjects. The collected blood cell samples were further subject to red blood cell lysis, followed by DNA extraction with QIAamp^®^ DNA Blood Mini Kit (Qiagen, CA, USA) and/or RNA extraction with mirVana^™^ miRNA Isolation Kit (Ambion, CA, USA). The RNA samples were converted into cDNA for qPCR assays and the DNA samples were treated with EZ DNA Methylation-Lightning^™^ Kit (Zymo Research, Irvine, USA) for bisulfite conversion. The detailed protocols were described in our previous study (Huang et al., 2018).

The bisulfite converted DNA samples were subject to genome-wide examination of DNA methylation with Infinium MethylationEPIC BeadChip (also called M850K assay, Illumina, CA, USA), which measured the methylation percentages (called beta values) of approximately 850,000 CpG dinucleotides. Only the DNA samples from three HC subjects and the DNA samples from all five AE subjects were subject to M850K assays. The DNA data of these eight samples include the eight samples (GSM3895259, GSM3895260, GSM3895261, GSM3895262, GSM3895263, GSM3895264, GSM3895265, GSM3895266) in NCBI GEO with accession number GSE132866.

### Cell Culture and Bead Enrichment of Fresh B Cells

In this study, we cultured two leukocyte cell lines and one BBB endothelial cell line. The first leukocyte cell line is HL-60 (Bioresource Collection and Research Center, BCRC No. 60027). HL-60 is a lymphoblast cell line derived from a 36 year-old Caucasian female with acute promyelocyte leukemia. It was cultured with a specified medium: 80% Iscove’s modified Dulbecco’s medium with 4 mM L-glutamine adjusted to contain 1.5 g/L sodium bicarbonate + 20% fetal bovine serum. HL-60 was differentiated into neutrophil-like cells with dimethyl sulfoxide treatment as described in a previous study (Huang et al., 2018). The second leukocyte cell line is Toledo (ATCC^®^ CRL-2631). Toledo is a B cell and was isolated from a white adult female with diffuse large cell lymphoma. It was cultured in the following medium: 90% RPMI 1640 medium with 2 mM L-glutamine adjusted to contain 1.5 g/L sodium bicarbonate, 4.5 g/L glucose, 10 mM HEPES, and 1.0 mM sodium pyruvate + 10% fetal bovine serum.

hCMEC (hCMEC/D3, Millipore Cat. #SCC066) is a BBB cell line derived from human temporal lobe microvessels and further immortalized by lentiviral vector. It was cultured with the VEGF-free EndoGRO-MV culture medium, which is specifically designed for culturing human microvascular endothelial cells. Fresh B cells from a healthy subject were also used in this study. They were isolated from total blood with CD19 antibody coated beads (DETACHaBEAD^®^ CD19 kit, Invitroen, CA, USA) as suggested by the manufacturer.

### Leukocyte Transendothelial Migration Assay

We conducted LTEM assay by referring to a previous study (Huang et al., 2018). In brief, 2 × 10^5^ hCMECs were first seeded into gelatin-coated hanging inserts (also called the upper chamber, Merck, NJ, USA) for 24 h. Then, the inserts were placed into 24-well culture plates (also called the lower chamber). The leukocyte cells (Toledo, HL-60 and fresh B cells) were first treated with 20% serum (from either HC or AE subjects) and/or recombinant proteins (S100A6 or S100A11) for 4 h.

S100A6 and S100A11 human recombinant proteins were both the products of ProSpec (ProSpec, Rehovot, Israel) with catalog number pro-148 and pro-385, respectively. S100A6 and S100A11 were both produced with *Escherichia coli*. The former has a molecular mass of 12.3 kDa with 20 amino acid His tag at the N-terminal. The latter has a molecular mass of 17kDa with an amino terminal hexahistidine tag. The purities of S100A6 and S100A11 human recombinant proteins are both greater than 95.0% as determined by SDS-PAGE. They were used to treat cells with the concentration of 10 µg/ml as suggested by a previous study (Huang et al., 2018).

Then, 1 × 10^5^ leukocyte cells were placed in the inserts for 2-h to allow infiltration through BBB endothelial layer. The leukocyte cells penetrating the BBB endothelial layer and migrating into the lower chamber were collected, stained with CD19-FITC (BD Biosciences) and quantified with the LSRII flow cytometry (BD Biosciences).

### ELISA

The ELISA method is usually applied to measure the concentrations of the proteins secreted to ex-cellular environment. We used the ARG82251 Human S100A6 ELISA Kit (Arigo biolaboratories, Hsinchu, Taiwan) to measure the concentrations of S100A6 in serum samples as suggested by the manufacturer’s protocol.

## Results

### Subject Information

In this study, we enrolled two male HC, two female HC, three male AE, and two female AE subjects. Their information about age and gender was tabulated in **Supplementary Table 1**. We first used the t-test and chi-square test to ensure that neither the age nor gender factor was significantly different between the HC and AE subjects. In addition, the clinical symptoms served as the AE diagnosis criteria and their incidence rates were also shown. The clinical and pathological data for the AE patients were tabulated in **Supplementary Table 1**.

### Flow Cytometry With CD19 Antibody Staining Distinguished B Lymphocytes From BBB Endothelial Cells

**Figure 1:**
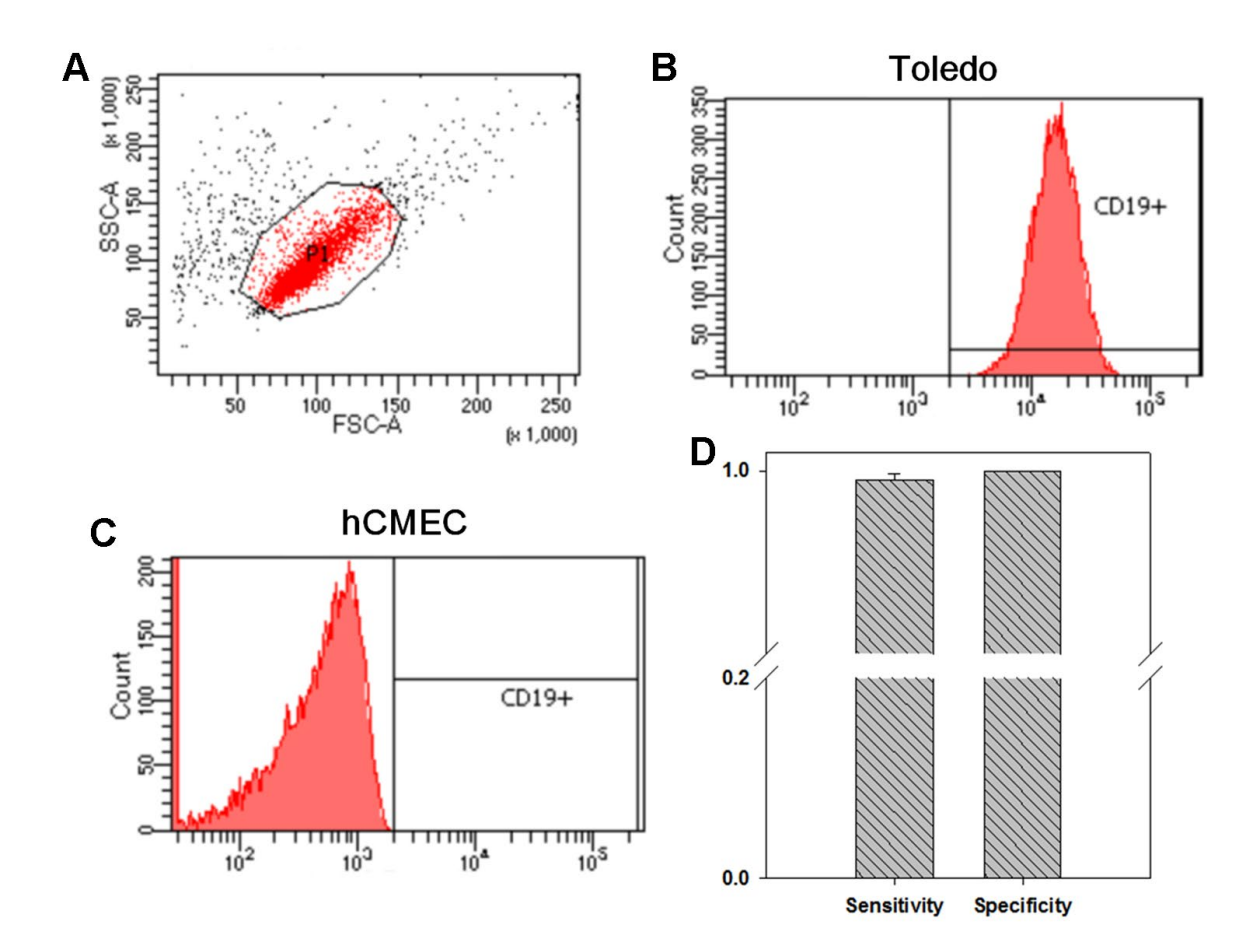
The investigation on whether flow cytometry with CD19 antibody staining distinguishes B cells from blood–brain barrier (BBB) endothelial cells. We investigated the distinguishability of flow cytometry on the two types of cells. **(A)** We first determined the specified FSC-A and SSC-A values which would be applied to gate the cells without treatment. **(B)** Among the 10,000 gated Toledo cells (one type of B cell), 9,965 ones were detected to be CD19^+^. **(C)** Among the 10,000 gated hCMEC cells (one type of BBB endothelial cell), no one was detected to be CD19^+^. **(D)** By three independent assays, in average, 9,954 Toledo cells and 0 hCMEC cells were detected to be CD19^+^, leading to a 99.54% sensitivity and a 100% specificity in recognizing B cell by CD19 antibody staining (N = 3). Data was presented as mean ± S.D.

In our previous study focusing on the pathogenesis of Kawasaki disease, we developed a customized LTEM assay to evaluate neutrophil infiltration through the coronary artery endothelial layer (Huang et al., 2018). Since AE is characterized by B cell infiltration through the BBB endothelial layer, we modified the *in vitro* LTEM assay for AE. We first examined whether B cells and BBB endothelial cells could be distinguished based on CD19 surface marker. Using flow cytometry, we first determined the target set of observed cells based on the specified FSC-A and SSC-A values (**Figure 1A**). Then, we succeeded in detecting 99.65% of the gated Toledo cells (one type of B cell line) with CD19-positive staining (**Figure 1B**). For the gated hCMEC BBB cells (one type of BBB endothelial cell), 0% was detected with the same CD19-positive staining (**Figure 1C**). As shown in **Figure 1D**, such CD19 antibody staining method had 99.54% sensitivity (for B cells) and 100.0% specificity (for hCMEC BBB cells) in recognizing B cells, enabled us to conduct further flow cytometry-based assays.

### AE Serum Specifically Promoted B Cells to Penetrate the BBB Endothelial Layer Without Affecting Neutrophils

**Figure 2:**
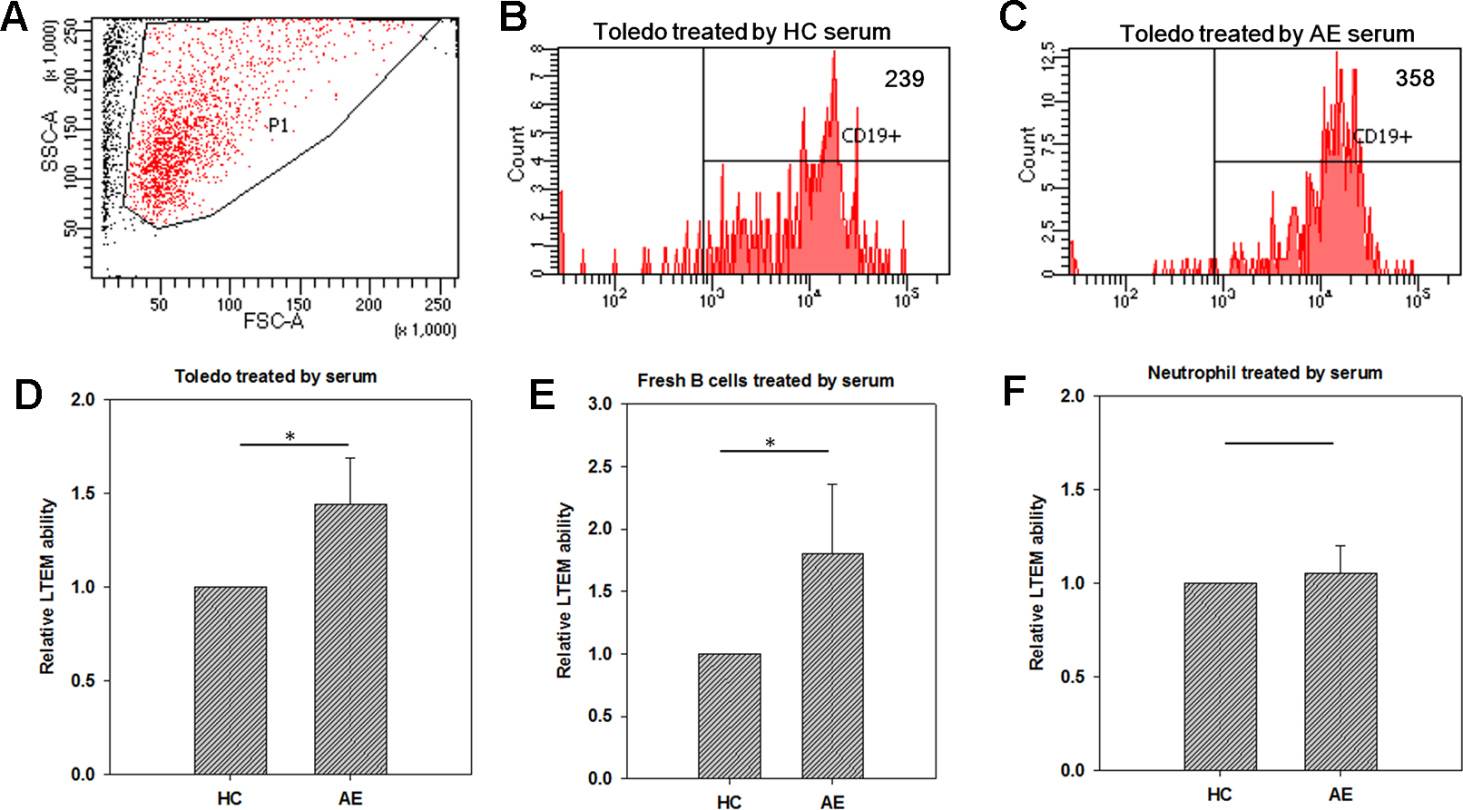
Using *in vitro* leukocyte transendothelial migration (LTEM) assay to investigate whether serum promotes leukocyte to penetrate BBB endothelial layer. We developed the specific LTEM model to study autoimmune encephalitis (AE) by making the endothelial layer with the cells of BBB endothelial cell line (hCMEC). **(A)** We first determined the specified FSC-A and SSC-A values which would be applied to gate the cells with LTEM assay. **(B)** When treated with HC serum, 239 of the treated Toledo cells were detected. **(C)** When the Toledo cells were treated with AE serum, we collected 358 ones. **(D)** AE serum significantly promoted Toledo cells to penetrate the BBB endothelial layer by almost 1.5 times than HC serum did (N = 4). **(E)** When fresh B cells were applied to replace Toledo cell, consistent and significant result was observed (N = 4). **(F)** No significant difference was observed when neutrophil-like cells were applied (N = 4). Data was presented as mean ± S.D. * denoted p-value < 0.05.

We further conducted LTEM assays on B cells treated with serum samples collected from HC subjects or AE patients. In a test run, we collected the B cells penetrating the BBB endothelial layer and respecified the FSC-A and SSC-A values of the target set of observed cells (**Figure 2A**). These specified FSC-A and SSC-A values were applied in all flow cytometry assays in this study. When treated with HC serum, 239 Toledo cells penetrated the BBB endothelial layer (**Figure 2B**). When treated with AE serum, the number of Toledo cells penetrating the BBB endothelial layer increased by 1.50-fold (**Figure 2C**). By using three independent assays, we found that AE serum significantly promoted Toledo cells to penetrate BBB endothelial layer compared with HC serum (**Figure 2D**).

Since Toledo is an immortalized tumor cell line, we also examined whether the promotion ability of AE serum can be replicated in the fresh B cells isolated from peripheral blood. To answer this question, we collected fresh B cells from the total blood of an HC subject with microbeads, followed by the same LTEM assays. It turned out that consistent and significant result was observed (**Figure 2E**), which further confirmed the promotion ability of AE serum as demonstrated by **Figure 2D**. We also examined whether neutrophil-like cells (differentiated from HL-60 with dimethyl sulfoxide treatment) could penetrate the BBB endothelial layer when treated with serum. As shown in **Figure 2F**, no significant difference was observed between the HC and AE serum treatments. In summary, the LTEM assays showed that AE serum, compared with HC serum, significantly and specifically promoted B cells to penetrate the BBB endothelial layer without affecting neutrophil cells.

### Genome-Wide Examination of DNA Methylation in Total White Blood Cell Samples

Although our current results demonstrated that AE serum promoted B lymphocyte infiltration, what crucial factors in serum contributed to this promotion ability is still unknown. Since DNA methylation played important roles in many autoimmune diseases, including systemic lupus erythematosus (Mok et al., 2016), type 1 diabetes (Kindt et al., 2018), rheumatoid arthritis (Miao et al., 2018), and so on, we also conducted a genome-wide examination of DNA methylation to search for potential disease genes. The DNA samples of total white blood cell from five AE patients and three HC subjects were examined with Infinium MethylationEPIC BeadChip (M850K assay). Among the eight subjects, three were female and the rest five ones were male. As a result, we had two factors in this M850K assay, namely gender (male vs. female) and disease (HC vs. AE). The raw data of M850K assay was first transformed by Illumina GenomeStudio software and further analyzed by Partek (Qiagen, Hilder, Germany) with default parameters specified. The significance criteria of a CpG dinucleotide were p-value < 0.05 and (Chang > 1.05 or Chang <−1.05).

We first compared the overall variations of DNA methylation caused by the two factors. As shown in **Supplementary Figure 1A**, the PCA plot showed that the gender factor separated the samples better than the disease factor, implying that male vs. female comparison generated more variations of DNA methylation than AE vs. HC comparison did. Such implication was confirmed by the “source of variation” plot (**Supplementary Figure 1B**) which was the output result of ANOVA. Therefore, the gender factor could not be ignored when identifying AE disease-related DNA methylation. For this concern, we enumerated the union (24,359 CpG dinucleotides in total) of the significant CpG dinucleotides based on the two factors, followed by heat map analysis. As shown in **Supplementary Figure 2**, the samples were mainly clustered into two sets, namely male and female. Such result was consistent with the conclusion of **Supplementary Figures 1A and B**. However, only a small fraction of CpG dinucleotides located within the intersection of the two factors (gender and disease), allowing us to exclude the CpG dinucleotides significant based on gender factor. As a result, the remaining 10,957 CpG dinucleotides were differentially methylated between AE and HC samples (**Supplementary Figure 1C** and **Supplementary Table 2**).

We further mapped the 10,956 significant CpG dinucleotides back to genome (hg19) and identified the genes regulated by these CpG dinucleotides based on the definition of a previous study (Huang et al., 2018). As a result, 6,571 CpG dinucleotides were located at the promoter regions of 6,582 genes. The information of these overlapping genes were available in **Supplementary Table 3**.

### S100A6 Was Hypo-Methylated and Up-Regulated in AE Patients

Among the 4,014 genes regulated by DNA methylation, we were especially interested in the members of the S100A gene family because they were deeply involved in inflammation-related diseases (Donato et al., 2013; Tong et al., 2014; Batycka-Baran et al., 2015). Based on our criteria, we identified three S100A family genes, including S100A5, S100A6, and S100A11.

**Figure 3:**
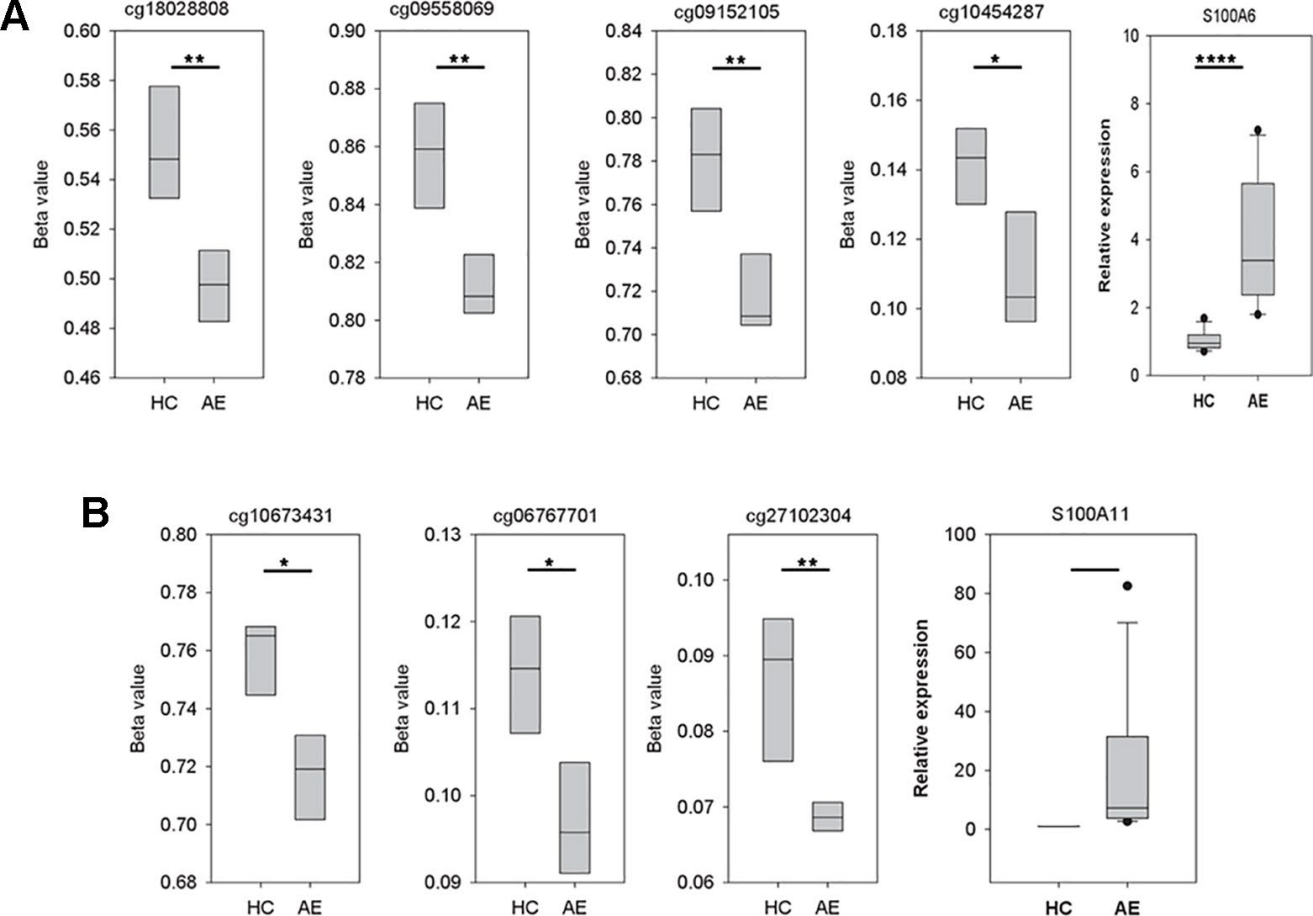
The variations of CpG dinucleotide methylation and gene expression for S100A6 and S100A11. The methylation profiles were determined with M850K assay and the gene expression data was produced by qPCR assay (five AE samples vs. four HC samples). The sequences of forward and reverse qPCR primers for S100A6 were TTCCACAAGTACTCCGGCA and ACCTCCTGGTCCTTGTT, respectively. For S100A11, they were ATTGCTGTCTTCCAGAAGTATG and TTGGTGTCCAGTTTCTTCATC. 18S was applied as internal control with forward and reverse qPCR primers as: GTAACCCGTTGAACCCCATT and CCATCCAATCGGTAGTAGCG. We performed real-time quantitative PCR using the Fast SYBR^®^ Green Master Mix system and the StepOnePlus™ System (Applied Biosystems). **(A)** The four CpG dinucleotides, cg18028808, cg09558069, cg09152105, and cg10454287, were in turn located at the −4556, −2178, −917, and −369 position of S100A6 promoter. **(B)** The three CpG dinucleotides, cg10673431, cg06767701, and cg27102304, were in turn located at the −598, 634, and 2122 position of S100A11 promoter. *, **, and **** denoted p-value < 0.05, 0.01, and 0.0001, respectively.

As illustrated in **Figure 3A**, there were four significant CpG dinucleotides at the promoter regions of S100A6 and they were all hypo-methylated in the AE samples. In addition, qPCR assays also showed that S100A6 was up-regulated in AE samples. Further correlation assays demonstrated a moderate negative correlation (Pearson’s correlation coefficient R = −0.65) between cg18028808 and S100A6. For the remaining three CpG dinucleotides, moderate negative correlations were also observed (R ranging from −0.58 to −0.66). Although S100A11 had three significant hypo-methylated CpG dinucleotides at its promoter or/and gene body regions, it was not significantly differentially expressed between HC and AE samples (**Figure 3B**). The correlation assay revealed modest negative correlations between the three CpG dinucleotides and S100A11 (R values ranging from −0.38 to −0.39). The detailed genomic coordinates and relative positions of these CpG dinucleotides were shown in **Table 1**.

**Table 1:** The genomic coordinates and relative positions of CpG dinucleotides examined.

CpG dinucleotide	Regulate gene: transcript	Genomic coordinate	Relative position to TSS
cg18028808	S100A6: NM_014624	153,513,274	−4556
cg09558069	S100A6: NM_014624	153,510,896	−2178
cg09152105	S100A6: NM_014624	153,509,635	−917
cg10454287	S100A6: NM_014624	153,509,087	−369
cg10673431	S100A11: NM_005620	152,010,110	−598
cg06767701	S100A11: NM_005620	152,008,878	634
cg27102304	S100A11: NM_005620	152,007,390	2122

We tabulated the genomic coordinates (hg19) of the examined CpG dinucleotides on the promoter regions of S100A6 and S100A11. Their positions relative to transcription start site (TSS) were also shown. S100A6 and S100A11 were both located at the minus strand of chromosome 1. The TSSs of NM_014624 and NM_005620 were 153,508,718 and 152,009,512, respectively. The CpG dinucleotides with minus “relative position to TSS” were located at the upstream of TSS, usually called promoter region. The CpG dinucleotides (cg06767701 and cg27102304) with plus “relative position to TSS” were located at the downstream of TSS, also called gene body.

### S100A6 Promoted B Cells to Penetrate the BBB Endothelial Layer

**Figure 4:**
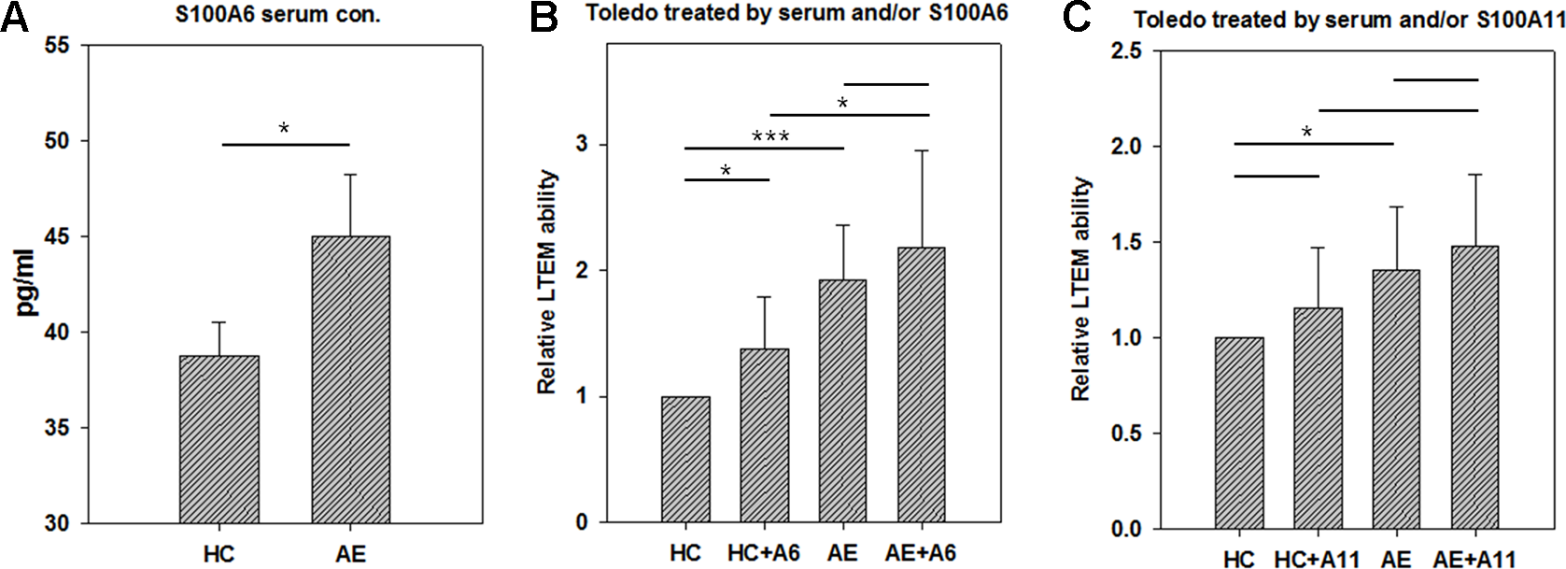
The results of *in vitro* LTEM assays on Toledo cells with serum and/or S100A recombinant protein treatment. To investigate whether serum and S100A proteins promote *in vitro* Toledo cell infiltration, we conducted AE-specified LTEM assays by making the BBB endothelial layer with hCMEC cells. **(A)** We conducted ELISA to examine serum level of S100A6 in four HC and five AE subjects. **(B)** The LTEM assay result of S100A6 (N = 4). **(C)** The LTEM assay result of S100A11 (N = 5). Data was presented as mean ± S.D. * and *** denoted p-value < 0.05 and 0.001, respectively.

To investigate the pathogenesis mechanism of S100A6 and A11 in AE, we conducted the LTEM assays with Toledo cells. S100A proteins are usually secreted into plasma in which they function as extra-cellular regulators. We conducted ELISA to examined the concentration of S100A6 in serum samples. As shown in **Figure 4A**, the AE serum samples had significantly higher S100A6 than HC serum samples did. Then, we treated Toledo cells with serum and/or S100A recombinant proteins. As shown in **Figures 4B and C**, AE serum significantly promoted B cell infiltration as previously shown in **Figure 2**. The addition of S100A6 protein significantly promoted B cells to infiltrate BBB endothelial layer both in HC and AE sera (**Figure 4B**). For S100A11, similar trend was observed but the promotion ability was not significant (**Figure 4C**).

## Discussion

AE is a potential reversible neurological disorder caused by the production of autoantibodies against neuronal receptors and synaptic proteins. It has been proposed that autoantibodies and/or activated B cells infiltration through the BBB endothelial layer is a key mechanism of AE. However, few AE-related studies have focused on the local leukocyte migration at the boundary of a blood vessel. A leukocyte transendothelial migration assay was used to investigate the local leukocyte migration at the boundary of a blood vessel (Huang et al., 2018) and to evaluate the severity of vascular inflammation (Walsh, 2009). In this study, we modified an *in vitro* LTEM model to demonstrate that AE serum promoted B cells to penetrate the barrier layer composed of BBB endothelial cells. Using this model, we demonstrated that the serum of AE patients increased the migration of B lymphocytes through BBB. For higher confidence and robust results, we applied not only the immortalized B cell line but also the freshly isolated B cells from a volunteer subject. Although the two types of cells demonstrated the consistent results, little difference could be observed that immortalized B cells reflected lower standard deviation than fresh B cells did (0.24 vs. 0.56). Further comparison showed that immortalized B cells and fresh B cells had coefficient of variations of 16.75% and 30.48%, respectively. Therefore, we applied immortalized B cell (Toledo cell) in most of the assays.

DNA methylation was usually investigated in autoimmune diseases. In patients with systemic lupus erythematosus, DNA hypo-methylation was observed in T cells and interferon-related genes (Richardson et al., 1990; Coit et al., 2013) and disease activity was associated with the amount of hypo-methylation in T cells (Richardson et al., 1990). Patients with rheumatoid arthritis show aberrant methylation in different type of T cells and hypo-methylation of B cells (Glossop et al., 2014). In addition, Kawasaki disease patients were also characterized with DNA hypo-methylation at the acute phase (Li et al., 2016). In our study, **Supplementary Figure 1C** also reflected the same phenomenon. This is in agreement with previous studies of autoimmune disorders. The hypo-methylated promoter regions are likely to activate down-stream immune-related genes, consequently contribute to the pathogenesis of AE.

S100A6 belongs to the S100A gene family, the protein products of which are capable of binding with Ca^2+^ (Tong et al., 2014). It was reported to be involved in cell proliferation, cytoskeletal dynamics, and tumorigenesis in many types tumors (Lesniak et al., 2017). S100A6 was shown to promote the proliferation of intrahepatic cholangiocarcinoma cells and of cervical cells *via* activating the p38/MAPK (Zheng et al., 2017) and PI3K/Akt pathways (Li et al., 2018), respectively. MiR-193a was reported to repress the oncogenesis reactions of lung cancer cells by directly targeting S100A6 (Li et al., 2019). In addition to oncogenesis roles, S100A6 also plays roles in many diseases. S100A6 could activate EGFR, triggering the downstream signaling reactions in HaCaT keratinocytes (Graczyk-Jarzynka et al., 2019). Its concentration in urine could also function as potential biomarkers of lupus nephritis activity (Turnier et al., 2017). Over-expression of S100A6 attenuated cardiomyocyte apoptosis and reduced infarct size in trasgenic mice (Mofid et al., 2017). In addition, S100A6 functioned as an extra-cellular regulator, modulating cell responses to stimuli (Donato et al., 2017). Therefore, we treated leukocytes with S100A6 recombinant proteins which highlighted its function as an extra-cellular regulator.

The major limitation of this study is the small sample size. In case–control studies, the more samples examined the more accurate results derived. In this study, only nine subjects were enrolled, including five AE subjects and four HC subjects. To compensate the limitation, we conducted detailed examinations of S100A6, including DNA methylation level by microarray, mRNA level by qPCR, and serum protein level by ELISA. By doing so, we expected to derive accurate results. The next limitation is the heterogeneous gender of subjects. Form the microarray cluster analysis (**Supplementary Figures 1** and **2**), we were surprised to find that gender factor contributed more methylation variation than disease factor. Therefore, in the future, we had better to eliminate the gender issue first. Namely, we should use the samples from the same gender on genome-wide screening assays, followed by specific validation assays on the samples of diverse sources.

In this study, we focused on the effects of serum and recombinant protein by comparing HC vs. AE and HC vs. HC + A6 (**Figure 4**). Actually, we were also interested in other comparisons. There was no significant difference between AE and AE + A6 treatments. Although S100A6 worked in the HC vs. HC+A6 comparison, it did not work in the AE vs. AE + A6 comparison. It seemed that the endogenous S100A6 reached a saturation concentration so that further addition of S100A6 recombinant proteins had less effect on B cells penetration. Another interesting comparison is HC + A6 vs. AE. Although no significant difference existed between HC + A6 and AE (p-value = 0.0524), AE seemed to cause higher relative LTEM ability. Actually S100A6 accounted for only a small fraction of AE serum and performed only part of the functions of AE serum. Therefore, S100A6 treatment in HC cannot fully recapitulate the effect of AE serum.

## Conclusion

In this study, we used AE patient serum and a new *in vitro* BBB model to demonstrate that antibody-producing B cells but not neutrophils are more likely to enter the CNS. A genome-wide methylation assay identified several hypo-methylated immune mediated genes, particularly S100A6 and S100A11, which are up-regulated in AE patients. The addition of recombinant S100A6 protein indeed increased the penetration of B lymphocytes through the BBB, which supports the role of S100A6 in AE. Further studies on how S100A6 protein facilitates B lymphocyte infiltration and whether other factors in AE serum also contribute to this phenomenon are likely to improve our understanding of AE, and hopefully to reveal novel therapeutic targets for this emerging treatable neurological disorder.

## Data Availability Statement

All methylation microarray data was submitted to NCBI GEO. Please refer to the accession number GSE132866.

## Ethics Statement

This study was approved by the institutional ethics board (IRB number: 201701437A3C601 and 201601451B0D001) and all subjects or their guardians signed the informed consent form.

## Author Contributions

M-HT and S-CL designed this study. C-HL, C-JH, and Y-TL were responsible for clinical sample collection and subject enrollment. YL, P-HL, K-WT, K-PW and M-HL conducted most of the assays. M-HT. C-HL, M-HL, and S-CL wrote this manuscript. S-CL supervised this work.

## Funding

This study was supported by the grants from Chang Gung Memorial Hospital (CMRPG8G1222 and CMRPG8G0131).

## Conflict of Interest

The authors declare that the research was conducted in the absence of any commercial or financial relationships that could be construed as a potential conflict of interest.

## Supplementary Material

Supplementary Figure 1 The result of DNA methylation examination with M850K assay. We had 3 male AE, 2 female AE, 2 male HC and 1 female HC DNA samples examined with M850K assay. Such design resulted in two-factor comparison, namely disease (AE vs. HC) and gender (male vs. female). We defined p-value<0.01 and variation ratio>10% as the criteria of significance, leading to 14,014 and 14,040 significant CpG dinucleotides identified in AE vs. HC and male vs. female comparisons, respectively. (a) The PCA plot showed that the gender factor separated the samples better than the disease factor. (b) Source of variation assay confirmed that that male vs. female comparison brought more variations of DNA methylation. (c) By removing the CpG dinucleotide simultaneously significant at both comparisons, the 11,645 CpG dinucleotides were clustered well based on disease factor. The red and green pixels denoted high and low beta value, respectively. As a result, most significant CpG dinucleotides were hypo-methylated in AE samples.

Supplementary Figure 2 The heat map of the union CpG dinucleotides. We collected the CpG dinucleotides simultaneously significant at both comparisons, forming a union of 24,359 CpG dinucleotides.

Supplementary Table 1 The clinical and pathological details of autoimmune encephalitis patients. We tabulated the clinical and pathological details of the AE patients. The average age of the four HC subjects was 44.50±15.15, resulting in a t-test p-value of 0.97 when comparing with the age of the AE subjects. Half of the HC subjects was female, resulting in a chi-square p-value of 0.76 when comparing with the gender of the AE subjects.

Supplementary Table 2 The details of CpG dinucleotides. The "?" sign denoted missing value. Such result could be the result of low-quality probe set signal.Such missing value were replaced with the mean of the same category. For example, cg00043673 has a missing value AE5-Beta, the remaining four AE sample have an average beta value of 0.2276. As a result, AE5-Beta is assigned as 0.2276.

Supplementary Table 3 The genes whose putative promoter regions were located by the significant CpG dinucleotides. We mapped the significant CpG dinucleotides back to genome then determined whether they were located at the putative promoter regions (from -5000 to +3000 bp of the transcription start site) of a transcript.
