# Supplementary figures and images for: S100A6 Promotes B Lymphocyte Penetration Through the Blood–Brain Barrier in Autoimmune Encephalitis
Source: Front Genet. 2019 Nov 22;10:1188. doi: 10.3389/fgene.2019.01188 (PMC6901080; doi:10.3389/fgene.2019.01188)

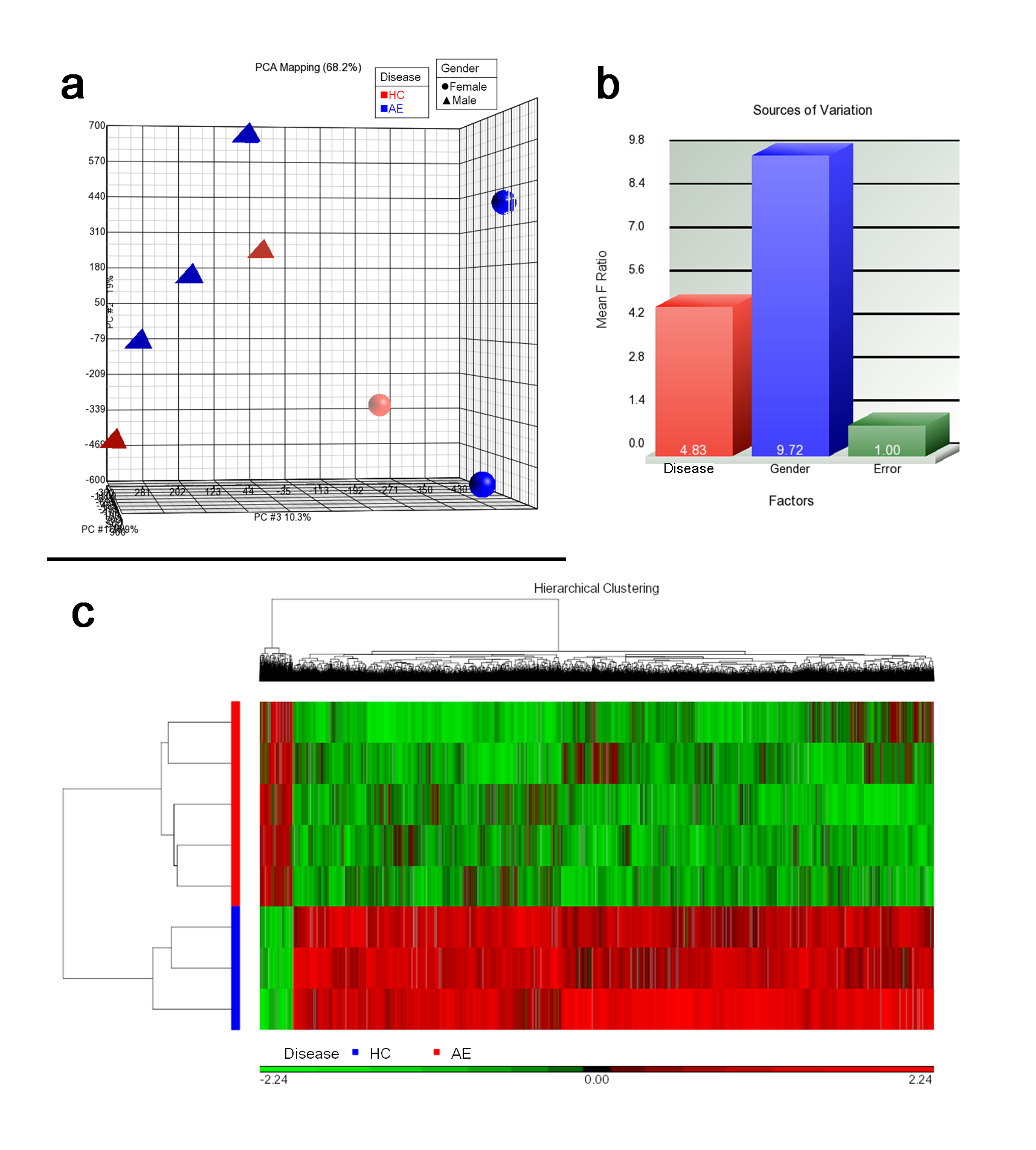

Supplement: Supplementary Figure 1 — The result of DNA methylation examination with M850K assay. We had 3 male AE, 2 female AE, 2 male HC and 1 female HC DNA samples examined with M850K assay. Such design resulted in two-factor comparison, namely disease (AE vs. HC) and gender (male vs. female). We defined p-value<0.01 and variation ratio>10% as the criteria of significance, leading to 14,014 and 14,040 significant CpG dinucleotides identified in AE vs. HC and male vs. female comparisons, respectively. (a) The PCA plot showed that the gender factor separated the samples better than the disease factor. (b) Source of variation assay confirmed that that male vs. female comparison brought more variations of DNA methylation. (c) By removing the CpG dinucleotide simultaneously significant at both comparisons, the 11,645 CpG dinucleotides were clustered well based on disease factor. The red and green pixels denoted high and low beta value, respectively. As a result, most significant CpG dinucleotides were hypo-methylated in AE samples. [file Image_1.tif]

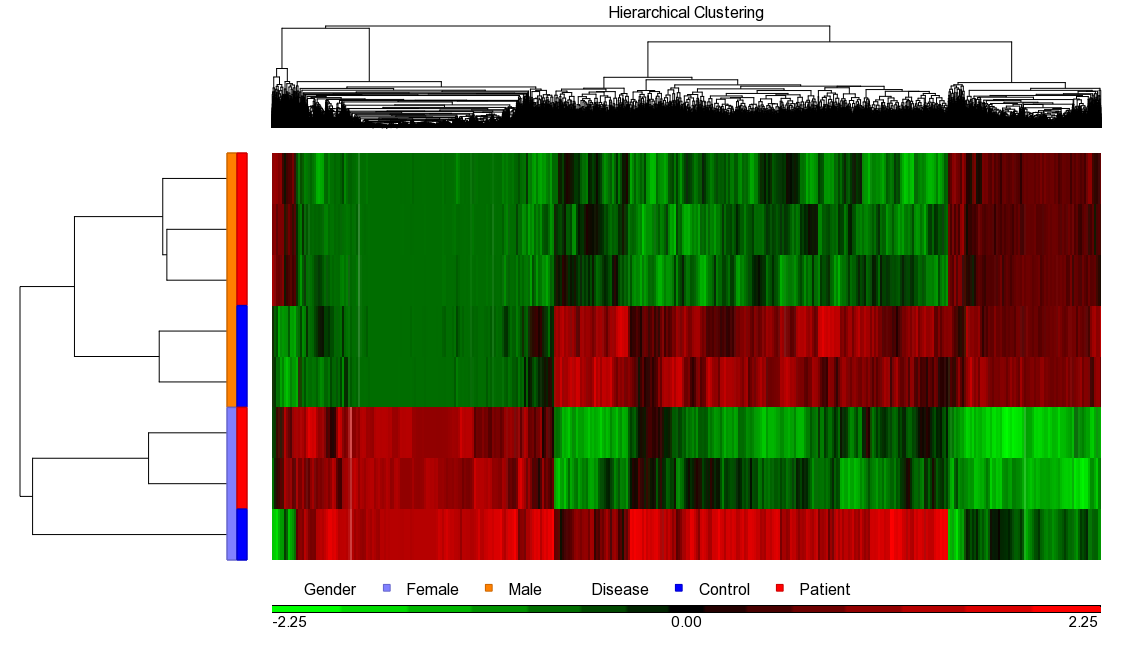

Supplement: Supplementary Figure 2 — The heat map of the union CpG dinucleotides. We collected the CpG dinucleotides simultaneously significant at both comparisons, forming a union of 24,359 CpG dinucleotides. [file Image_2.png]
