# Supplementary material for: S100A6 Promotes B Lymphocyte Penetration Through the Blood–Brain Barrier in Autoimmune Encephalitis
Source: Front Genet. 2019 Nov 22;10:1188. doi: 10.3389/fgene.2019.01188 (PMC6901080; doi:10.3389/fgene.2019.01188)
Supplement: Supplementary Table 1 — The clinical and pathological details of autoimmune encephalitis patients. We tabulated the clinical and pathological details of the AE patients. The average age of the four HC subjects was 44.50±15.15, resulting in a t-test p-value of 0.97 when comparing with the age of the AE subjects. Half of the HC subjects was female, resulting in a chi-square p-value of 0.76 when comparing with the gender of the AE subjects. [file Table_1.doc]

**Supplementary Table 1** **The clinical and pathological details of autoimmune encephalitis patients**. We tabulated the clinical and pathological details of the AE patients. The average age of the four HC subjects was 44.50±15.15, resulting in a t-test p-value of 0.97 when comparing with the age of the AE subjects. Half of the HC subjects was female, resulting in a chi-square p-value of 0.76 when comparing with the gender of the AE subjects.

| Case | Sex | Onset age | Duration from onset to receiving immunotherapy | Neurological symptoms | Seizure & SE type | MRI findings | Tumor screen | Ab Screening | Immunotherapy | Type of autoimmune encephalitis |
| --- | --- | --- | --- | --- | --- | --- | --- | --- | --- | --- |
| I | M | 63 | 7 days | Encephalitis with cognitive decline and recurrent episodes of NCSE | FMS with IA, BTCS  EPC, NCSE | Old hemorrhage over left parieto-occipital lobe. | No tumor found | Negative for anti- NMDA, AMPA, LGI1, CASPR2, and GABA_B_ receptors | IV MP  IV cyclophosphamide  IT rituximab | AE with unknown antigen |
| II | F | 79 | 39 days | Encephalitis with seizure | FMS | Old resorbed hematoma at right basal ganglia. | No tumor found | Negative for anti- NMDA, AMPA, LGI1, CASPR2, and GABA_B_ receptors | Oral MP | AE with unknown antigen |
| III | F | 24 | 5 days | Encephalitis with involuntary movement | Focal hyperkinetic seizure with IA  Seizure clusters | Hyperintensity of bilateral caudate nucleus and putamen in DWI imaging | No tumor found | Anti-NMDA Ab (+) | IV MP  IT rituximab | Anti-NMDAR Encephalopathy |
| IV | M | 26 | 6 days | Encephalitis with super refractory status epilepticus. Coma for one month | FMS with IA, EPC, NCSE | Hyperintensity of left mesial temporal lobe in FLAIR imaging | No tumor found | Negative for anti- NMDA, AMPA, LGI1, CASPR2, and GABA_B_ receptors | IV MP  IV rituximab  IT rituximab  plasmapheresis | AE with unknown antigen |
| V | M | 28 | 12 days | Encephalitis with status epilepticus | FMS with IA, BTCS | Hyperintensity of bilateral mesial temporal lobe in FLAIR imaging | No tumor found | Anti-NMDA Ab (+) | IV MP  plasmapheresis | Anti-NMDAR Encephalopathy |

Abbreviations: AE = autoimmune encephalitis; BTCS = bilateral tonic-clonic seizure**;** EPC = epilepsia partialis continua; F = female; FMS = focal motor seizure; IA = impaired awareness; IT = intrathecal; IV = intravenous; M = male; MP= methylprednisolone; NCSE = nonconvulsive status epilepticus; SE = status epilepticus.
